# Evaluation of risk factor management of patients treated on an internal nephrology ward: a pilot study

**DOI:** 10.1186/1472-6904-9-15

**Published:** 2009-09-06

**Authors:** Gunar Stemer, Sonja Zehetmayer, Rosa Lemmens-Gruber

**Affiliations:** 1 Department of Pharmacology and Toxicology, University of Vienna, Althanstraße 14, 1090 Vienna, Austria; 2 Pharmacy Department, Vienna General Hospital, Währinger Gürtel 18-20, 1090 Vienna, Austria; 3 Department of Medical Statistics, Medical University of Vienna, Spitalgasse 23, 1090 Vienna, Austria

## Abstract

**Background:**

The objectives of this pilot study were to evaluate treatment quality for the risk factors of hypertension, diabetes and hyperlipidemia as well as the overall treatment quality for patients on an internal nephrology ward. This evaluation included the collection of data concerning the quality of therapeutic drug monitoring, drug use and potential drug-drug interactions. Establishing such baseline information highlights areas that have a need for further therapeutic intervention and creates a foundation for improving patient care, a subject that could be addressed in future clinical pharmacy research projects.

**Methods:**

Medical charts of patients treated on a single internal nephrology ward were retrospectively evaluated using a predefined data collection form. Assessment of further need for therapeutic intervention was performed.

**Results:**

For 76.5% (n = 78) of the total study population (n = 102), there was either a possibility (39.2%, n = 40) or a need (37.3%, n = 38) for further intervention based on the overall assessment. For the risk factors of hypertension, diabetes and hyperlipidemia, the proportions of patients that require further intervention were 78.8% (n = 71), 90.6% (n = 58) and 87.9% (n = 58), respectively. Patients with diabetes or hyperlipidemia were less likely to have optimal risk factor control. The number of drugs prescribed and the number of potential drug-drug interactions were significantly higher after in-hospital treatment.

**Conclusion:**

Risk factor treatment needs optimisation. Risk factor management, systematic medication reviews, and screening for and management of potential drug-drug interactions deserve great attention. Clinical pharmacy services could help in the achievement of treatment goals.

## Background

Health-care professionals, such as physicians, nurses, and (clinical) pharmacists, in both inpatient and outpatient settings are increasingly confronted with a growing number of patients with chronic kidney disease (CKD) and end-stage renal disease (ESRD)[1]. Medical care for CKD patients is complex due to widespread co-morbidities and major risk factors (RF) for CKD or cardiovascular disease (CVD) [2,3]. The progression of CKD and the deterioration of kidney function from stage 1 CKD [3] to more severe stages can be slowed by optimal treatment of underlying co-morbidities and RFs, which can be accomplished with lifestyle modifications and/or different pharmacological interventions that address the treatment of hypertension, diabetes mellitus and hyperlipidemia, among others. The slowing down of disease progression is pivotal for prolonging the period before stage 5 CKD or ESRD, which involves the necessary initiation of either dialysis or evaluation of suitability for kidney transplantation. Several initiation and progression factors have been shown to influence disease onset and progression [3,4]. Large-scale efforts that target these RFs have been initiated to improve outcomes in the CKD population [5].

The involvement of clinical pharmacists as members of the interdisciplinary patient care team responsible for the management of many different diseases has proven to be beneficial and has been associated with positive patient outcomes [6-8]. Clinical pharmacists have also been influential in the field of nephrology and have provided valuable support for the achievement of defined goals in the treatment of different RFs and management of drug-related problems in the ESRD population [9-12].

This pilot study was performed to establish baseline data that address (1) the quality of RF management, (2) overall treatment quality, (3) quality of therapeutic drug monitoring (TDM), (4) quantitative drug use at admission and discharge and (5) the frequency of potential drug-drug interactions (pDDIs) in the studied patient population as well as in the predefined subgroup of kidney transplant patients (TX subgroup). The retrospective evaluation of these parameters should identify areas with the need for further intervention and possibilities for the improvement of patient care that could be addressed in future clinical pharmacy research.

## Methods

### Study design, group and setting

A retrospective review was conducted of 102 randomly selected medical histories of patients receiving treatment between August 2006 and April 2008 on an internal nephrology ward of General Hospital in Vienna. Data were collected between January and May 2008. There were no direct interventions performed on patients. This descriptive study was approved by the local ethics committee of the Medical University of Vienna and the Vienna General Hospital.

### Data sources and collection

Medical charts, physicians' admission and discharge letters and cumulative laboratory findings were the only data sources used. Data were collected according to a predefined data collection form, which was divided into six categories: (1) sociodemographic criteria; (2) cause of hospitalisation, further medical conditions (co-morbidities) and underlying renal disease; (3) treatment of the predefined RFs of hypertension, diabetes mellitus and hyperlipidemia in the total population and quality of TDM in the TX subgroup; (4) drug regimen at the time of admission and discharge; (5) number and severity of pDDIs and (6) overall quality of RF treatment. Furthermore, glomerular filtration rates (GFRs) at discharge and at admission were estimated using the Modification of Diet in Renal Disease (MDRD) study equation. Stages of CKD (based on GFR at discharge) were assigned according to the National Kidney Foundation/Kidney Disease Outcomes Quality Initiative (NKF/KDOQI) classification [3].

### Assessment of RF treatment quality and overall assessment

Treatment quality during hospitalisation was assessed according to established guidelines for each RF, for quality of TDM in the TX subgroup and for overall treatment quality (see Table 1). The quality of RF and TDM management as well as overall treatment quality was assessed numerically on a scale from one to four (see Table 2). Patient treatment histories that were assessed as being a two or three on this quality scale were compiled and categorised as patients for whom further therapeutic intervention would have been either beneficial (2) or necessary (3) and therefore would represent potential domains for intervention by a clinical pharmacist.

**Table 1:** Risk factor reference values

**Risk factor**	**Reference Values**
Hypertension ^23,34^	Non-diabetic patients <140/90 mm/Hg
	Diabetic patients <130/80 mm/Hg
	Patients with diabetic nephropathy <125/75 mm/Hg
Diabetes mellitus ^34^	Fasting plasma blood glucose <110 mg/dl
	Glycosylated haemoglobin HbA1c 4-6%
Hyperlipidemia ^25^	Low density cholesterol <130 mg/dl
	Total cholesterol <200 mg/dl
	Triglycerides <200 mg/dl

**Table 2:** Categories for assessment of individual risk factors, therapeutic drug monitoring and overall assessment

	**Individual RF^**a **^and TDM**^**b**^		**Assessment of overall treatment quality**	
	
	**Assessment**	**Explanation**	**Assessment**	**Explanation**
**1**	No need for intervention	Values^d ^according to references in more than 2/3 of available values; Values better at discharge than at admission; Disease/RF^a ^is treated; no severe pDDIs^c^	Very good RF^a ^management	No improvements necessary
**2**	Improvement possible	Values^d ^outside of reference range in more than 1/3 of available values; Values worse at discharge; severe pDDIs^c^; RF is treated	Good RF^a ^management	Up to two individual RFs^a ^being assessed as "improvement possible" (category 2); no untreated RFs^a ^(category 3)
**3**	Disease untreated	No drug therapy for RF^a ^treatment; no TDM performed, although appropriate	Improvement in RF^a ^management needed	More than two individual RFs^a ^or TDMs^b ^being assessed as "improvement possible" (category 2) or untreated RF^a ^(category 3)
**4**	No conclusion possible	Missing data; inconclusive data	No conclusion possible	Missing data; inconclusive data

^a ^RF = risk factor

^b ^TDM = therapeutic drug monitoring

^c ^pDDI = potential drug-drug interaction

^d ^e.g., blood pressure, fasting blood glucose, lipid levels, plasma levels of immunosuppressants

### Screening for pDDIs

Admission drug histories and discharge drug histories were electronically screened for pDDIs using Medis^®^. pDDIs were classified into four categories of relevance given by the database, namely *severe*, *moderate*, *minor *and *unknown relevance *(see Appendix for detailed explanations). Only pDDIs classified as *severe *and *moderate *were included in the statistical analysis. Individual drug dosages were not taken into account when assessing pDDIs.

### Statistical analysis

Absolute and relative frequencies as well as 95% confidence intervals (lower CI and upper CI) are reported for the four categories of overall assessment for each RF and for overall RF management. Statistical analyses were conducted on the total study population and for the TX subgroup. To analyse the influence of the RFs (hypertension, diabetes mellitus and hyperlipidemia) on assessment category, an ordinal logistic regression analysis of assessment categories one, two or three (see Table 2) was calculated (category 4 is omitted). The probability of the patient being in a higher category was also modelled. P-values, odds ratios and the corresponding 95% confidence intervals are given. For the analysis, the RFs of diabetes and hyperlipidemia were both classified into "no diabetes mellitus" or "no hyperlipidemia" versus "diabetes mellitus" or "hyperlipidemia". The analysis was performed using SAS 9. Means will be presented as mean (range, standard deviation).

## Results

### Sociodemographic and patient characteristics

**Figure 1:**
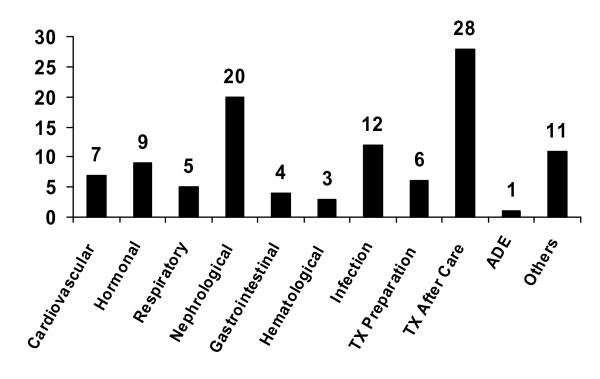
**Major causes of hospitalization, classified**. TX transplantation. ADE adverse drug event.

**Figure 2:**
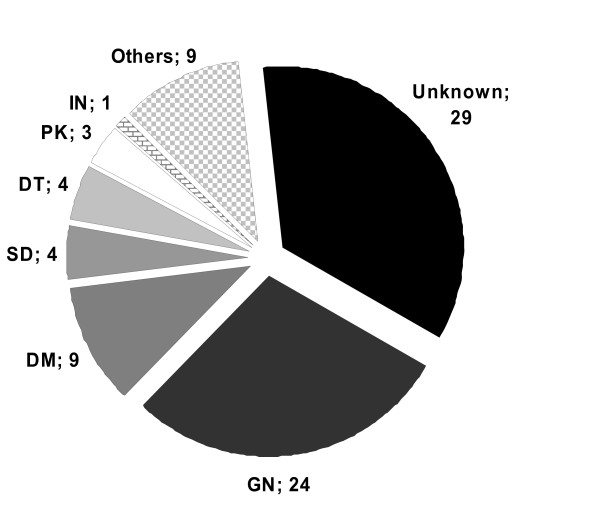
**Underlying nephrologic disease (where available)**. GN glomerulonephritis. DM diabetes mellitus. SD systemic diseases. DT drug toxicity. PK polycystic kidney. IN interstitial nephritis.

Sociodemographic characteristics and stages of CKD for the total study population and TX subgroup are shown in Table 3. Major causes of hospitalisation in the study population and the underlying renal diseases are shown in Figures 1 and 2, respectively.

**Table 3:** Sociodemographic characteristics, stages of CKD and length of stay

	**Total population n = 102**		**TX^**a **^subgroup n = 49**	
	
	**n**	**%**	**n**	**%**
Men/Women	67/35	65.7/34.3	37/12	75.5/24.5
Age, years				
Mean ± SD^b^	55.5 ± 13.4		55.4 ± 11.4	
Range	24-86		29-73	
BMI^c^, kg/m^2^				
Mean ± SD^b^	26.3 ± 5.1		26 ± 4.8	
Range	15-40.2		16-40.2	
Stages of CKD	n = 80		n = 44	
2	3	3.8	2	2.3
3	39	48.8	32	72.7
4	15	18.8	7	15.9
5	23	28.8	3	6.8
Length of stay, days				
Mean ± SD^b^	14.8 ± 10.5		17.06 ± 9.9	
Range	2-47		2-41	

^a ^TX = transplantation

^b ^SD = standard deviation

^c ^BMI = body mass index

### RF: hypertension

A diagnosis of hypertension was seen in 88.2% (90) of patients. The absolute and relative frequencies as well as the corresponding confidence intervals for hypertension are given in Table 4 for the four different categories of overall assessment of the total study population. In 78.8% (71) of patient cases, there was a possibility or need for further therapeutic interventions. Hypertensive patients were treated on the ward for a mean time of 15.2 days (d) (range 2-47, standard deviation 10.84), with an average of 7.7 d (0-45, 9.87) of blood pressure values out of the individual reference range. Estimation of renal function at admission and discharge showed a mean GFR of 23.1 and 30.1 mL/min/1.73 m^2^, respectively. Stages 2 to 5 CKD were present in 4.1, 50.7, 17.8 and 27.4% of hypertensive patients, respectively.

**Table 4:** Assessment of individual risk factors and quality of therapeutic drug monitoring

	**No need for intervention**		**Improvement possible**		**Disease untreated/** **No TDM**^a^		**No conclusion possible**	
	**% (n)**	**95% CI**^**b**^	**% (n)**	**95% CI**^**b**^	**% (n)**	**95% CI**^**b**^	**% (n)**	**95% CI**^**b**^
Hypertension n = 90	17.8 (16)	*0.10-0.26*	37.8 (34)	*0.28-0.48*	41.1 (37)	*0.31-0.51*	3.3 (3)	*0.00-0.07*
Diabetes mellitus n = 64	7.8 (5)	*0.01-0.14*	42.2 (27)	*0.30-0.54*	48.4 (31)	*0.36-0.61*	1.6 (1)	*0.00-0.05*
Hyperlipidemia n = 66	9.1 (6)	*0.02-0.16*	42.4 (28)	*0.31-0.54*	45.5 (30)	*0.33-0.57*	3.0 (2)	*0.00-0.07*
TDM^a^ n = 44	29.6 (13)	*0.17-0.45*	34.1 (15)	*0.20-0.50*	0.0 (0)	*-*	36.4 (16)	*0.22-0.52*

For explanations of assessment categories see table 2.

^a ^TDM = therapeutic drug monitoring

^b ^CI = confidence interval

### RF: diabetes mellitus and elevated fasting blood glucose

A total of 62.8% (64) of patients in this RF group had either a definitive diagnosis of diabetes (diabetes mellitus type I (3.9%, n = 4) or diabetes mellitus type II (30.39%, n = 31)) or continuously elevated fasting blood glucose (FBG) out of reference range (28.4%, n = 29). Absolute and relative frequencies as well as corresponding confidence intervals for the four different categories of overall assessment for the total study population are given in Table 4. The majority (90.6%, 58) of patients had a need for further therapeutic intervention. Patients with diabetes mellitus type I were treated on the ward for an average of 11.3 d (4-22, 8.14), with FBG levels out of reference range on 5.5 d (1-10, 4.65). Patients with diabetes mellitus type II were treated for an average of 30.0 d (2-45, 11.9), with FBG levels out of reference on 6.9 d (1-24, 6.36). Patients with continuously elevated FBG levels were treated for 16.4 d (2-39, 9.89), with elevated FBG for 6.7 d (2-19, 4.6) on average. Glycosylated haemoglobin (HbA1_c_) levels were evaluated and analysed as a marker of long-term treatment quality. In 43.8% (n = 28) of patients in the diabetes RF group, there was no information available about HbA1_c _values. In 25% (n = 16) of patients, reported HbA1_c _levels were in accordance with the reference range (see Table 1), and in 25% (n = 16) of patients HbA1_c _levels were outside of the reference range. Of patients with HbA1_c_values outside of the reference range, 68.8% (n = 11) had diabetes type II. Estimation of renal function at admission and discharge showed a mean GFR of 23.2 and 30.2 mL/min/1.73 m^2^, respectively. Stages 2 to 5 CKD were present in 3.9, 49.0, 21.6 and 25.5% of patients with the RF of diabetes, respectively.

### RF: Hyperlipidemia

Of the patients reviewed, 64.7% (n = 66) were diagnosed with hyperlipidemia, while 41.2% (n = 42) showed continuously elevated cholesterol-levels and 5.9% (n = 6) showed elevated triglyceride-levels. HMG-Co-enzyme-A-inhibitors (statins) were used in 17.7% (18) of patients for cardiovascular event prophylaxis. Absolute and relative frequencies as well as corresponding confidence intervals of the hyperlipidemia RFs for the four different categories of overall assessment for the total study population are given in Table 4. A possible need for further therapeutic intervention was found in 87.9% (58) of the patients in the study. Estimation of renal function at admission and discharge showed a mean GFR of 21.7 and 28.4 mL/min/1.73 m^2^, respectively. Stages 2 to 5 CKD were present in 1.9, 49.1, 20.8 and 28.3% of patients with the RF of hyperlipidemia, respectively.

### Characteristics and quality of TDM

The plasma drug levels of immunosuppressant medications were determined and the dosages were adjusted in 89.8% (44) of the TX subgroup patients. Immunosuppressive medications primarily consisted of a three-way combination of calcineurin inhibitors (tacrolimus (79.6%, 35) or ciclosporin (20.5%, 8)), anti-metabolites (mycophenolate mofetil (70.5%, 35), mycophenolic acid (18.2%, 8) or azathioprine (4.5%, 2)) and corticosteroids. In 25% (11) of the TX subgroup patients, a switch in immunosuppressant medication was necessary due to adverse drug events (ADEs). For example, tacrolimus induced tremors and mycophenolate mofetil induced diarrhoea. The quality of TDM was only assessable if a defined therapeutic range was available in the medical chart (61.4%, 27). The number of days with sub-therapeutic and supra-therapeutic concentrations was evaluated based on these defined ranges. Absolute and relative frequencies of TDM for the four different categories of overall assessment in the TX subgroup are given in Table 4.

### Overall assessment of treatment quality

Absolute and relative frequencies and corresponding confidence intervals for overall assessment of treatment quality in the total study population and TX subgroup are shown in Table 5. A need for further optimisation of RF treatment was observed in 76.5% (78) of the total study population and 81.6% (40) of the TX subgroup.

**Table 5:** Overall assessment of treatment quality

	**Very good RF^**a **^management**		**Good RF^**a **^management**		**Improvement needed**		**No conclusion possible**	
	**% (n)**	**95% CI**^**b**^	**% (n)**	**95% CI**^**b**^	**% (n)**	**95% CI**^**b**^	**% (n)**	**95% CI**^**b**^
Total n = 102	19.6 (20)	*0.12-0.27*	39.2 (40)	*0.30-0.49*	37.3 (38)	*0.28-0.47*	3.9 (4)	*0.00-0.08*
TX^c ^subgroup n = 49	16.3 (8)	*0.06-0.27*	32.7 (16)	*0.20-0.46*	49.0 (24)	*0.35-0.63*	2.0 (1)	*0.00-0.06*

For explanations of assessment categories see table 2.

^a ^RF = risk factor

^b ^CI = confidence interval

^c ^TX = transplantation

### Influence of individual RFs on overall treatment quality

Regression analysis showed that the diabetes mellitus and hyperlipidemia RFs had a significant impact on assessment outcome. Patients with diabetes (p = 0.001, OR 4.309, 95%CI: 1.81-10.25) or hyperlipidemia (p = 0.0085, OR 3.146, 95%CI: 1.34-7.39) had a higher overall risk of being assessed in category 2 (*good risk factor management, but improvement possible*) or category 3 (*improvement needed*). This correlation was not shown for the hypertension RF (p = 0.2704, OR 2.056, 95%CI: 0.57-7.40).

### Quantitative drug use and pDDIs in the total study population

The total sum of prescribed drugs in the total study population was 1110 at admission and 1220 at discharge. Table 6 shows the number of drugs prescribed, number of pDDIs and number of pDDIs per drug prescribed.

**Table 6:** Quantitative drug use and potential drug-drug interactions at hospital admission and discharge

	**Admission**		**Discharge**		
**Total study population n = 102**	**Mean ± SD**^**a**^	**Range**	**Mean ± SD**^**a**^	**Range**	**P-value**^**b**^
Number of drugs per patient	10.9 ± 4.2	0-20	12.1 ± 4.3	2-21	<0.0001*
Number of pDDIs^c ^per patient	1.9 ± 1.9	0-8	2.7 ± 2.5	0-11	<0.0001*
Number of pDDIs^c ^per drug prescribed	0.2 ± 0.2	0-0.83	0.2 ± 0.2	0-0.64	0.0016*
TX^d ^subgroup n = 49					

Number of drugs per patient	12.6 ± 3.1	4-20	13.3 ± 3.2	5-20	0.055
Number of pDDIs^c ^per patient	1.8 ± 2.4	0-8	2.7 ± 2.8	0-11	0.014*
Number of pDDIs^c ^per drug prescribed	0.1 ± 0.1	0-0.53	0.2 ± 0.2	0-0,64	0.014*

Only pDDIs classified as moderate or severe were included in the analysis.

^a ^SD = standard deviation

^b ^p = statistical significance according to the t-test

^c ^pDDIs = potential drug-drug interactions

^d ^TX = transplantation

* Statistically significant

All three parameters showed significantly higher values at discharge compared to admission. Treatment on the ward was significantly associated with an elevated number of drugs prescribed and an elevated number of pDDIs.

In the total study population, 45.1% (46) of patients had an increase in the number of pDDIs during treatment on the ward, 41.2% (42) had no change in the number of pDDIs and 13.7% (14) had a decrease in the number of pDDIs. In 43.2% (44) of all evaluated patients, at least one pDDI was associated with an increased probability for nephrotoxicity, thus increasing the risk of acute renal failure and aggravation of renal function.

### Quantitative drug use and pDDIs in the TX subgroup

The sum of drugs prescribed to the TX subgroup patients was 619 at admission, compared with 650 at time of discharge. The number of drugs prescribed, number of pDDIs and number of pDDIs per drug prescribed are shown in Table 6.

In-hospital treatment was associated with a significantly elevated number of pDDIs per patient and pDDIs per drug prescribed. When the number of drugs prescribed per patient was compared, there was no statistically significant difference. In 44.9% (22) of the TX subgroup patients, the number of pDDIs increased during treatment on the ward, 38.8% (19) of patients had no change in the number of pDDIs and 16.3% (8) of patients had a decreased incidence of pDDIs during treatment on the ward. In 83.7% (41) of evaluated patients, at least one pDDI was associated with an increased probability of nephrotoxicity, which increased those patients' risk of developing acute renal failure and having an aggravation of renal function.

## Discussion

The study results show that the management of the individual RFs of hypertension, diabetes and hyperlipidemia requires improvement. In the overall assessment of treatment quality, more than three-quarters of the patients showed a possibility or evident need for further intervention to reach the treatment goals. Very good RF management was evident in less than 20% of patients for each of the investigated RFs. For diabetes and hyperlipidemia, this proportion was even under the 10% threshold. Based on regression analysis, patients with diabetes or hyperlipidemia were four and three times less likely, respectively, to have optimal RF control. Our results are consistent with published studies and reviews that address treatment quality and adherence to treatment guidelines for hypertension [13-19], diabetes mellitus [15,20,21] and hyperlipidemia [15,19] in CKD patients.

The apparent need for improvement in RF control in our study population must be discussed in light of the special features of the nephrological patient population.

Hypertension, either as a cause or a complication of CKD, is prevalent in up to 75% of patients with CKD stage 3-5, in up to 80% of kidney transplant patients and in up to 90% of maintenance haemodialysis patients [22,23]. Virtually all patients in the study population had kidney function of CKD stage 3 or worse, nearly 50% had one or more kidney transplantations performed, and 27% were dependent on renal replacement therapy (e.g., haemo- or peritoneal dialysis). The very high prevalence and the multifactorial pathogenesis of hypertension in renal disease (e.g., sodium retention and fluid overload and structural kidney changes) and the steady decline in renal function make it difficult *per se *to reach tight treatment goals. Antihypertensive polypharmacotherapy was therefore almost necessary in our study population to even approximate treatment goals. Our study findings stress the importance of drawing attention to tight blood pressure control, as in about the half of the treatment period, blood pressure control was suboptimal. Second, control of diabetes and hyperlipidemia management was also suboptimal. The relevance of these findings is emphasised by the fact that diabetes is not only the leading cause of CKD in developed countries [24], but diabetes and hyperlipidemia are also two of the most important RFs for cardiovascular disease. Of note, CKD patients represent *a priori *the highest risk group for CVD [3]. Therefore, guidelines [24,25] recommend strict glycemic and lipidemic control. Besides patients with a confirmed diagnosis of diabetes mellitus, we also included patients with continuously elevated FBG in the diabetes RF group. Continuously elevated FBG represents, in itself, a RF for the development of diabetes mellitus II, and therefore, clarification and management deserves attention. One fourth of patients in the diabetes RF group had glycosylated haemoglobin values outside of the reference range, confirming the need for improvement of long-term glycemic control, especially for diabetes mellitus II where around 68% of patients had HbA1_c _levels outside of the reference range. In nearly 50% of patients in the diabetic subgroup, glycosylated haemoglobin values were totally lacking, and therefore, no information was available concerning the long-term control of their diabetes. Furthermore, the proportion of untreated hyperlipidemia of around 45% also stresses the need for intervention and improvement. Nearly half of our study population was kidney transplant patients. Thus, concomitant immunosuppressive therapy may have also negatively biased RF control, as hypertension, diabetes and hyperlipidemia are all well-described side effects of calcineurin inhibitors. However, our study was not designed to assess a potential correlation. Finally, the main focus during hospitalisation often lies in curing acute disease and in necessary treatment, and consequently, optimisation of RF treatment often takes a back seat. Simple negligence and unintended oversight may also be considered as reasons for suboptimal RF control. In summary, there seems to be vast room for improvement in the control of the investigated RFs in our study population. Clinical pharmacists' activities have proved beneficial for the achievement of treatment goals [10-12].

Our study also examined the quality of TDM in patients receiving immunosuppressants. For the quality analysis, the number of TDM drug levels outside of the reference range was used as a surrogate parameter. For approximately 40% of patients, written information regarding the desired drug concentration range, depending on time since transplantation, was missing in the medical charts and therefore could not be assessed. It was found that only approximately one third of patients with kidney transplants were without need of further intervention. This assessment emphasises the fact that immunosuppressant dose adjustments are common and optimal dosing regimens are difficult to determine, especially in the early postoperative phase [26,27]. Furthermore, frequent medication changes, namely drug additions and discontinuations, complicate dosing regimen optimisation. Widely-used immunosuppressives have great inter- and intra-individual pharmacokinetic variability and many confounding factors (e.g., race, time since transplantation, sex and metabolic profile) that have to be taken into account when adapting dosages on the basis of plasma drug concentration [28]. Constant plasma drug levels corresponding to time since transplantation should be the goal. ADEs are also common in the kidney transplant patient population. Common ADEs seen with immunosuppressives are as follows: new-onset diabetes mellitus, tremors (tacrolimus), hyperlipidemia, hypertension, hypertrichosis (ciclosporin), and gastrointestinal side effects, such as diarrhoea (mycophenolate mofetil) [27]. Typical management of ADEs considers dose reduction of the offending drug or switching to another immunosuppressant medication. All these properties impair dose adjustments and tight drug-level control of immunosuppressant medications. There is evidence that clinical pharmacists can contribute to the vigilant supervision and management of kidney transplant patients [9,29,30].

Evaluation of drug use on the nephrology ward shows that in-hospital treatment is associated with a significant increase in the number of prescribed drugs and pDDIs. Poly-morbidity is frequent, and multiple medications are almost always necessary to meet treatment goals. Our study illustrates that poly-medication, which is almost inevitable in nephrology patients, leads to an increasing number of pDDIs. Other authors report similar findings in other patient populations [31,32]. It must be noted that the number of drugs administered to the patient during the active in-hospital treatment period is even higher compared to the number at admission or discharge due to temporary therapeutic treatments, such as anti-infectives or anticoagulation drugs. Reviewing drug-drug interactions at admission and discharge provides only a fractional view of all pDDIs that by definition can never be complete. According to a published study by Glintborg and colleagues, the clinical relevance of computerised screening of pDDIs, as done in our study, tends to be low [33]. However, in daily practice, this tool proves to be useful for gaining a quick overview and raising awareness of potential medication-related events. Considering the sensitivity of patients with renal impairment and drug-related needs, especially for pDDIs leading to increased nephrotoxicity or aggravation of kidney function, these interactions must be intensely and carefully monitored. Recognition, avoidance and management of drug-drug interactions and medication reviews should be done vigilantly [3] as these procedures also represent markers of treatment quality.

This pilot study was retrospective and was primarily designed to identify different areas with intervention needs (e.g., RFs, TDM) and possibilities for improvement of drug therapy-related aspects (e.g., management of pDDIs, medication reviews). Evidence from the literature shows that these tasks are already performed by clinical pharmacists as a part of their clinical routine. However, the extent of clinical pharmacists' involvement varies considerably. We are aware that this pilot study itself does not contribute to the overall evidence on clinical pharmacy services. However, we hypothesise that clinical pharmacists could play an important part in improving treatment quality, as there is evidence supporting the benefit of clinical pharmacy services in this area [7-11]. Since the process of delivering drug therapy to in-hospital patients is a complex, time-consuming, multi-step and therefore error-prone process, clinical pharmacy services could enforce drug-therapy safety and address therapeutic needs that are being insufficiently met by other health care professionals in the care delivery process.

As with all studies, our current investigations had limitations. The assessment was done by a single pharmacist and included only patients from one internal nephrology ward. Data from other wards were not available. Therefore, the possibility of data extrapolation is limited.

## Conclusion

Our pilot study identifies possibilities and needs for improvements in the management of hypertension, diabetes and hyperlipidemia, which are three major RFs for renal and/or CV disease. In the subgroup of TX patients, tight control of immunosuppressant blood levels according to the reference range could be optimised. Medication regimens are complex, and the frequency of pDDIs increased during in-hospital treatment. Detected pDDIs were frequently associated with a potential aggravation of already impaired kidney function. Clinical pharmacy services could positively influence RF management, TDM and the management of pDDIs. However, this hypothesis must be confirmed in future research. Based on our study findings, the impact of clinical pharmacy services on drug-therapy related problems and RF management should be addressed using a prospective study design in a nephrology patient population and a kidney transplant population, respectively.

## Competing interests

The study was performed as part of a clinical pharmacy project that was funded by Amgen. The authors declare that there are no financial or other conflicts of interests with respect to the contents of the article.

## Authors' contributions

GS was responsible for the study design, data collection and interpretation and preparation of the manuscript. SZ was responsible for the study design, statistical analysis of collected data and reviewing the manuscript. RLG was responsible for study design, data interpretation and reviewing the manuscript. All authors read and approved the final manuscript.

## Appendix

Medis^® ^is an Austrian general drug information tool with a pDDIs screening function. The data used originates from Mikropharm - Arzneimittelinteraktionen provided by a collaboration of the Bundesvereinigung Deutscher Apothekerverbände (ABDA), Österreichische Apothekerkammer (ÖAK) and Schweizer Apothekerverein (SAV).

The four categories of relevance were:

Severe interaction: combination may be life threatening; possibility of intoxication; permanent damage may be induced.

Moderate interaction: combination may lead to therapeutic difficulties and may even be harmful; close patient monitoring is needed.

Minor interaction: interaction is to be taken into account but normally causes no harm to the patient.

Unknown relevance: no proven clinical relevance of described interaction.

## Pre-publication history

The pre-publication history for this paper can be accessed here:


